# Supplementary material for: The impact of COVID-19 lockdown on nursing higher education at Chengdu University
Source: PLoS One. 2023 Jun 16;18(6):e0286290. doi: 10.1371/journal.pone.0286290 (PMC10275429; doi:10.1371/journal.pone.0286290)
Supplement: S1 File — (PDF) [file pone.0286290.s001.pdf]

No.	Sex	Class	Year	D.O.B	Age
1	Female	Nursing (bachalor)18-1	2019	1998	21
2	Female	Nursing (bachalor)18-1	2019	1998	21
3	Female	Nursing (bachalor)18-1	2019	1998	21
4	Female	Nursing (bachalor)18-1	2019	2000	19
5	Female	Nursing (bachalor)18-1	2019	1998	21
6	Female	Nursing (bachalor)18-1	2019	2000	19
7	Female	Nursing (bachalor)18-1	2019	1999	20
8	Female	Nursing (bachalor)18-1	2019	1999	20
9	Female	Nursing (bachalor)18-1	2019	1995	24
10	Female	Nursing (bachalor)18-1	2019	2000	19
11	Female	Nursing (bachalor)18-1	2019	1998	21
12	Female	Nursing (bachalor)18-1	2019	1998	21
13	Female	Nursing (bachalor)18-1	2019	1999	20
14	Female	Nursing (bachalor)18-1	2019	1999	20
15	Female	Nursing (bachalor)18-1	2019	2000	19
16	Female	Nursing (bachalor)18-1	2019	2000	19
17	Female	Nursing (bachalor)18-1	2019	1999	20
18	Female	Nursing (bachalor)18-1	2019	1999	20
19	Female	Nursing (bachalor)18-1	2019	1998	21
20	Female	Nursing (bachalor)18-1	2019	2000	19
21	Female	Nursing (bachalor)18-1	2019	1996	23
22	Male	Nursing (bachalor)18-1	2019	1998	21
23	Male	Nursing (bachalor)18-1	2019	2000	19
24	Male	Nursing (bachalor)18-1	2019	1998	21
25	Male	Nursing (bachalor)18-1	2019	1999	20
26	Female	Nursing (bachalor)18-2	2019	2000	19
27	Male	Nursing (bachalor)18-2	2019	2000	19
28	Female	Nursing (bachalor)18-2	2019	1998	21
29	Female	Nursing (bachalor)18-2	2019	2000	19
30	Female	Nursing (bachalor)18-2	2019	2001	18
31	Female	Nursing (bachalor)18-2	2019	1999	20
32	Female	Nursing (bachalor)18-2	2019	1999	20
33	Female	Nursing (bachalor)18-2	2019	2000	19
34	Female	Nursing (bachalor)18-2	2019	1996	23
35	Female	Nursing (bachalor)18-2	2019	1997	22
36	Female	Nursing (bachalor)18-2	2019	2000	19
37	Female	Nursing (bachalor)18-2	2019	2000	19
38	Female	Nursing (bachalor)18-2	2019	1998	21
39	Female	Nursing (bachalor)18-2	2019	2000	19
40	Female	Nursing (bachalor)18-2	2019	1999	20
41	Female	Nursing (bachalor)18-2	2019	1999	20
42	Female	Nursing (bachalor)18-2	2019	2000	19
43	Female	Nursing (bachalor)18-2	2019	1998	21

44	Male	Nursing (bachalor)18-2	2019	1999	20
45	Male	Nursing (bachalor)18-2	2019	2000	19
46	Male	Nursing (bachalor)18-2	2019	1999	20
47	Male	Nursing (bachalor)18-2	2019	2000	19
48	Male	Nursing (bachalor)18-2	2019	2000	19
49	Male	Nursing (bachalor)18-2	2019	2001	18
50	Male	Nursing (bachalor)18-2	2019	2000	19
51	Female	Nursing (bachalor)18-3	2019	2000	19
52	Female	Nursing (bachalor)18-3	2019	1999	20
53	Female	Nursing (bachalor)18-3	2019	1997	22
54	Female	Nursing (bachalor)18-3	2019	1997	22
55	Female	Nursing (bachalor)18-3	2019	2000	19
56	Female	Nursing (bachalor)18-3	2019	1998	21
57	Female	Nursing (bachalor)18-3	2019	1997	22
58	Female	Nursing (bachalor)18-3	2019	2000	19
59	Female	Nursing (bachalor)18-3	2019	1999	20
60	Female	Nursing (bachalor)18-3	2019	1998	21
61	Female	Nursing (bachalor)18-3	2019	1998	21
62	Female	Nursing (bachalor)18-3	2019	2000	19
63	Female	Nursing (bachalor)18-3	2019	1999	20
64	Female	Nursing (bachalor)18-3	2019	1999	20
65	Female	Nursing (bachalor)18-3	2019	1997	22
66	Female	Nursing (bachalor)18-3	2019	1999	20
67	Female	Nursing (bachalor)18-3	2019	2000	19
68	Male	Nursing (bachalor)18-3	2019	1998	21
69	Male	Nursing (bachalor)18-3	2019	1999	20
70	Male	Nursing (bachalor)18-3	2019	1998	21
71	Male	Nursing (bachalor)18-3	2019	1999	20
72	Male	Nursing (bachalor)18-3	2019	2001	18
73	Male	Nursing (bachalor)18-3	2019	1995	24
74	Female	Nursing (bachalor)18-4	2019	1996	23
75	Female	Nursing (bachalor)18-4	2019	1999	20
76	Female	Nursing (bachalor)18-4	2019	1998	21
77	Female	Nursing (bachalor)18-4	2019	2000	19
78	Female	Nursing (bachalor)18-4	2019	2000	19
79	Female	Nursing (bachalor)18-4	2019	1999	20
80	Female	Nursing (bachalor)18-4	2019	1999	20
81	Female	Nursing (bachalor)18-4	2019	2000	19
82	Female	Nursing (bachalor)18-4	2019	2000	19
83	Female	Nursing (bachalor)18-4	2019	1997	22
84	Female	Nursing (bachalor)18-4	2019	1999	20
85	Female	Nursing (bachalor)18-4	2019	1998	21
86	Female	Nursing (bachalor)18-4	2019	1998	21
87	Female	Nursing (bachalor)18-4	2019	1999	20
88	Female	Nursing (bachalor)18-4	2019	2000	19

89	Female	Nursing (bachalor)18-4	2019	1998	21
90	Female	Nursing (bachalor)18-4	2019	1999	20
91	Female	Nursing (bachalor)18-4	2019	2000	19
92	Female	Nursing (bachalor)18-4	2019	1999	20
93	Female	Nursing (bachalor)18-4	2019	1999	20
94	Male	Nursing (bachalor)18-4	2019	1999	20
95	Male	Nursing (bachalor)18-4	2019	2000	19
96	Male	Nursing (bachalor)18-4	2019	1999	20
97	Male	Nursing (bachalor)18-4	2019	1999	20
98	Male	Nursing (bachalor)18-4	2019	1999	20
99	Male	Nursing (bachalor)18-4	2019	1998	21
100	Female	Nursing (bachalor)18-5	2019	1999	20
101	Female	Nursing (bachalor)18-5	2019	1997	22
102	Female	Nursing (bachalor)18-5	2019	1999	20
103	Female	Nursing (bachalor)18-5	2019	1997	22
104	Female	Nursing (bachalor)18-5	2019	2000	19
105	Female	Nursing (bachalor)18-5	2019	1999	20
106	Female	Nursing (bachalor)18-5	2019	1993	26
107	Female	Nursing (bachalor)18-5	2019	1999	20
108	Female	Nursing (bachalor)18-5	2019	1999	20
109	Female	Nursing (bachalor)18-5	2019	1999	20
110	Female	Nursing (bachalor)18-5	2019	1999	20
111	Female	Nursing (bachalor)18-5	2019	1996	23
112	Female	Nursing (bachalor)18-5	2019	2000	19
113	Female	Nursing (bachalor)18-5	2019	2001	18
114	Female	Nursing (bachalor)18-5	2019	2000	19
115	Female	Nursing (bachalor)18-5	2019	1999	20
116	Female	Nursing (bachalor)18-5	2019	1997	22
117	Female	Nursing (bachalor)18-5	2019	2000	19
118	Male	Nursing (bachalor)18-5	2019	1998	21
119	Male	Nursing (bachalor)18-5	2019	2000	19
120	Male	Nursing (bachalor)18-5	2019	2000	19
121	Male	Nursing (bachalor)18-5	2019	2000	19
122	Male	Nursing (bachalor)18-5	2019	2000	19
123	Male	Nursing (bachalor)18-5	2019	2001	18
124	Male	Nursing (bachalor)18-5	2019	2000	19
125	Male	Nursing (bachalor)18-5	2019	1999	20
126	Female	Nursing (bachalor)18-6	2019	2000	19
127	Female	Nursing (bachalor)18-6	2019	1994	25
128	Female	Nursing (bachalor)18-6	2019	2000	19
129	Female	Nursing (bachalor)18-6	2019	1999	20
130	Female	Nursing (bachalor)18-6	2019	1998	21
131	Female	Nursing (bachalor)18-6	2019	2000	19
132	Female	Nursing (bachalor)18-6	2019	2000	19
133	Female	Nursing (bachalor)18-6	2019	1999	20

134	Female	Nursing (bachalor)18-6	2019	2000	19
135	Female	Nursing (bachalor)18-6	2019	2000	19
136	Female	Nursing (bachalor)18-6	2019	1998	21
137	Female	Nursing (bachalor)18-6	2019	1996	23
138	Female	Nursing (bachalor)18-6	2019	2000	19
139	Female	Nursing (bachalor)18-6	2019	1999	20
140	Female	Nursing (bachalor)18-6	2019	1997	22
141	Female	Nursing (bachalor)18-6	2019	2000	19
142	Female	Nursing (bachalor)18-6	2019	2000	19
143	Female	Nursing (bachalor)18-6	2019	1994	25
144	Female	Nursing (bachalor)18-6	2019	2000	19
145	Male	Nursing (bachalor)18-6	2019	1999	20
146	Male	Nursing (bachalor)18-6	2019	1999	20
147	Male	Nursing (bachalor)18-6	2019	1999	20
148	Male	Nursing (bachalor)18-6	2019	1999	20
149	Female	Nursing (bachalor)18-7	2019	1999	20
150	Female	Nursing (bachalor)18-7	2019	1998	21
151	Female	Nursing (bachalor)18-7	2019	1999	20
152	Female	Nursing (bachalor)18-7	2019	1999	20
153	Female	Nursing (bachalor)18-7	2019	1999	20
154	Female	Nursing (bachalor)18-7	2019	1998	20
155	Female	Nursing (bachalor)18-7	2019	1999	20
156	Female	Nursing (bachalor)18-7	2019	1999	20
157	Female	Nursing (bachalor)18-7	2019	1998	21
158	Female	Nursing (bachalor)18-7	2019	1999	20
159	Female	Nursing (bachalor)18-7	2019	2000	19
160	Female	Nursing (bachalor)18-7	2019	1999	20
161	Female	Nursing (bachalor)18-7	2019	2000	19
162	Female	Nursing (bachalor)18-7	2019	2000	19
163	Female	Nursing (bachalor)18-7	2019	1997	22
164	Female	Nursing (bachalor)18-7	2019	2001	18
165	Male	Nursing (bachalor)18-7	2019	2000	19
166	Male	Nursing (bachalor)18-7	2019	2000	19
167	Male	Nursing (bachalor)18-7	2019	1999	20
168	Male	Nursing (bachalor)18-7	2019	1999	20
169	Male	Nursing (bachalor)18-7	2019	1999	20
170	Male	Nursing (bachalor)18-7	2019	2000	19
171	Male	Nursing (bachalor)18-7	2019	1997	22
172	Female	Nursing (bachalor)18-8	2019	1998	21
173	Female	Nursing (bachalor)18-8	2019	2000	19
174	Female	Nursing (bachalor)18-8	2019	2000	19
175	Female	Nursing (bachalor)18-8	2019	1998	21
176	Female	Nursing (bachalor)18-8	2019	2000	19
177	Female	Nursing (bachalor)18-8	2019	1998	21
178	Female	Nursing (bachalor)18-8	2019	2000	19

179	Female	Nursing (bachalor)18-8	2019	1999	20
180	Female	Nursing (bachalor)18-8	2019	2000	19
181	Female	Nursing (bachalor)18-8	2019	1998	21
182	Female	Nursing (bachalor)18-8	2019	1998	21
183	Female	Nursing (bachalor)18-8	2019	2000	19
184	Female	Nursing (bachalor)18-8	2019	2000	19
185	Female	Nursing (bachalor)18-8	2019	1999	20
186	Female	Nursing (bachalor)18-8	2019	1999	20
187	Female	Nursing (bachalor)18-8	2019	1999	20
188	Female	Nursing (bachalor)18-8	2019	1999	20
189	Female	Nursing (bachalor)18-8	2019	2000	19
190	Male	Nursing (bachalor)18-8	2019	1999	20
191	Male	Nursing (bachalor)18-8	2019	1996	23
192	Male	Nursing (bachalor)18-8	2019	2000	19
193	Male	Nursing (bachalor)18-8	2019	2000	19
194	Male	Nursing (bachalor)18-8	2019	1998	21
195	Male	Nursing (bachalor)18-8	2019	1999	20
1	Female	Nursing (bachalor)19-1	2020	2000	20
2	Female	Nursing (bachalor)19-1	2020	2001	19
3	Female	Nursing (bachalor)19-1	2020	2001	19
4	Female	Nursing (bachalor)19-1	2020	2000	20
5	Female	Nursing (bachalor)19-1	2020	2001	19
6	Male	Nursing (bachalor)19-1	2020	2002	18
7	Female	Nursing (bachalor)19-1	2020	2000	20
8	Female	Nursing (bachalor)19-1	2020	2000	20
9	Male	Nursing (bachalor)19-1	2020	2000	20
10	Male	Nursing (bachalor)19-1	2020	1999	21
11	Male	Nursing (bachalor)19-1	2020	2000	20
12	Female	Nursing (bachalor)19-1	2020	2000	20
13	Female	Nursing (bachalor)19-1	2020	2000	20
14	Female	Nursing (bachalor)19-1	2020	2000	20
15	Female	Nursing (bachalor)19-1	2020	2002	18
16	Male	Nursing (bachalor)19-1	2020	2001	19
17	Female	Nursing (bachalor)19-1	2020	2001	19
18	Female	Nursing (bachalor)19-1	2020	2001	19
19	Male	Nursing (bachalor)19-2	2020	2000	20
20	Female	Nursing (bachalor)19-2	2020	2001	19
21	Female	Nursing (bachalor)19-2	2020	2000	20
22	Male	Nursing (bachalor)19-2	2020	2000	20
23	Female	Nursing (bachalor)19-2	2020	2000	20
24	Female	Nursing (bachalor)19-2	2020	2001	19
25	Male	Nursing (bachalor)19-2	2020	2000	20
26	Female	Nursing (bachalor)19-2	2020	2001	19
27	Male	Nursing (bachalor)19-2	2020	2001	19
28	Male	Nursing (bachalor)19-2	2020	2000	20

29	Female	Nursing (bachalor)19-2	2020	2000	20
30	Female	Nursing (bachalor)19-2	2020	2001	19
31	Female	Nursing (bachalor)19-2	2020	2000	20
32	Female	Nursing (bachalor)19-2	2020	2000	20
33	Female	Nursing (bachalor)19-2	2020	2001	19
34	Female	Nursing (bachalor)19-2	2020	2000	20
35	Female	Nursing (bachalor)19-2	2020	2001	19
36	Female	Nursing (bachalor)19-3	2020	2001	19
37	Female	Nursing (bachalor)19-3	2020	2002	18
38	Female	Nursing (bachalor)19-3	2020	2001	19
39	Female	Nursing (bachalor)19-3	2020	2000	20
40	Male	Nursing (bachalor)19-3	2020	2000	20
41	Female	Nursing (bachalor)19-3	2020	2001	19
42	Female	Nursing (bachalor)19-3	2020	2001	19
43	Male	Nursing (bachalor)19-3	2020	1999	21
44	Female	Nursing (bachalor)19-3	2020	2000	20
45	Male	Nursing (bachalor)19-3	2020	2001	19
46	Female	Nursing (bachalor)19-3	2020	2000	20
47	Female	Nursing (bachalor)19-3	2020	2000	20
48	Female	Nursing (bachalor)19-3	2020	2001	19
49	Male	Nursing (bachalor)19-3	2020	2000	20
50	Female	Nursing (bachalor)19-3	2020	2001	19
51	Female	Nursing (bachalor)19-3	2020	1999	21
52	Male	Nursing (bachalor)19-3	2020	2001	19
53	Male	Nursing (bachalor)19-3	2020	1999	21
54	Female	Nursing (bachalor)19-3	2020	2001	19
55	Female	Nursing (bachalor)19-3	2020	2000	20
56	Female	Nursing (bachalor)19-3	2020	2000	20
57	Female	Nursing (bachalor)19-4	2020	2001	19
58	Female	Nursing (bachalor)19-4	2020	2000	20
59	Male	Nursing (bachalor)19-4	2020	1998	22
60	Female	Nursing (bachalor)19-4	2020	2000	20
61	Female	Nursing (bachalor)19-4	2020	1998	22
62	Male	Nursing (bachalor)19-4	2020	1993	27
63	Male	Nursing (bachalor)19-4	2020	2001	19
64	Male	Nursing (bachalor)19-4	2020	1999	21
65	Female	Nursing (bachalor)19-4	2020	1995	25
66	Female	Nursing (bachalor)19-4	2020	1998	22
67	Female	Nursing (bachalor)19-4	2020	2001	19
68	Female	Nursing (bachalor)19-4	2020	2001	19
69	Female	Nursing (bachalor)19-4	2020	2001	19
70	Female	Nursing (bachalor)19-4	2020	2001	19
71	Female	Nursing (bachalor)19-4	2020	2001	19
72	Female	Nursing (bachalor)19-4	2020	2001	19
73	Female	Nursing (bachalor)19-5	2020	2001	19

74	Female	Nursing (bachalor)19-5	2020	2000	20
75	Female	Nursing (bachalor)19-5	2020	2001	19
76	Male	Nursing (bachalor)19-5	2020	2001	19
77	Female	Nursing (bachalor)19-5	2020	2001	19
78	Female	Nursing (bachalor)19-5	2020	2001	19
79	Female	Nursing (bachalor)19-5	2020	2001	19
80	Female	Nursing (bachalor)19-5	2020	2001	19
81	Female	Nursing (bachalor)19-5	2020	1998	22
82	Female	Nursing (bachalor)19-5	2020	2001	19
83	Female	Nursing (bachalor)19-5	2020	2000	20
84	Female	Nursing (bachalor)19-5	2020	2001	19
85	Female	Nursing (bachalor)19-5	2020	2000	20
86	Female	Nursing (bachalor)19-5	2020	1998	22
87	Female	Nursing (bachalor)19-5	2020	1999	21
88	Male	Nursing (bachalor)19-5	2020	1999	21
89	Female	Nursing (bachalor)19-5	2020	2001	19
90	Female	Nursing (bachalor)19-5	2020	1998	22
91	Female	Nursing (bachalor)19-5	2020	1999	21
92	Female	Nursing (bachalor)19-5	2020	1998	22
93	Female	Nursing (bachalor)19-5	2020	2000	20
94	Female	Nursing (bachalor)19-5	2020	2001	19
95	Female	Nursing (bachalor)19-5	2020	1998	22
96	Female	Nursing (bachalor)19-5	2020	1997	23
97	Male	Nursing (bachalor)19-5	2020	1998	22
98	Female	Nursing (bachalor)19-5	2020	1992	28
99	Male	Nursing (bachalor)19-5	2020	2000	20
100	Female	Nursing (bachalor)19-5	2020	2000	20
101	Female	Nursing (bachalor)19-5	2020	2001	19
102	Female	Nursing (bachalor)19-5	2020	2001	19
103	Female	Nursing (bachalor)19-5	2020	2000	20
104	Female	Nursing (bachalor)19-5	2020	2000	20
105	Male	Nursing (bachalor)19-5	2020	2000	20
106	Female	Nursing (bachalor)19-5	2020	2000	20
107	Female	Nursing (bachalor)19-5	2020	1999	21
108	Female	Nursing (bachalor)19-5	2020	2001	19
109	Female	Nursing (bachalor)19-6	2020	2001	19
110	Male	Nursing (bachalor)19-6	2020	1998	22
111	Female	Nursing (bachalor)19-6	2020	1999	21
112	Female	Nursing (bachalor)19-6	2020	2000	20
113	Female	Nursing (bachalor)19-6	2020	2001	19
114	Female	Nursing (bachalor)19-6	2020	2001	19
115	Female	Nursing (bachalor)19-6	2020	2000	20
116	Female	Nursing (bachalor)19-6	2020	1999	21
117	Female	Nursing (bachalor)19-6	2020	2000	20
118	Female	Nursing (bachalor)19-6	2020	1999	21

119	Female	Nursing (bachalor)19-6	2020	2002	18
120	Male	Nursing (bachalor)19-6	2020	1996	24
121	Male	Nursing (bachalor)19-6	2020	2001	19
122	Female	Nursing (bachalor)19-6	2020	2000	20
123	Female	Nursing (bachalor)19-6	2020	1997	23
124	Female	Nursing (bachalor)19-6	2020	1999	21
125	Male	Nursing (bachalor)19-6	2020	1997	23
126	Female	Nursing (bachalor)19-6	2020	1999	21
127	Female	Nursing (bachalor)19-6	2020	1999	21
128	Female	Nursing (bachalor)19-6	2020	2001	19
129	Female	Nursing (bachalor)19-6	2020	2000	20
130	Female	Nursing (bachalor)19-6	2020	2001	19
131	Female	Nursing (bachalor)19-6	2020	2001	19
132	Female	Nursing (bachalor)19-6	2020	2003	17
133	Female	Nursing (bachalor)19-6	2020	2001	19
134	Female	Nursing (bachalor)19-6	2020	2000	20
135	Female	Nursing (bachalor)19-6	2020	2001	19
136	Female	Nursing (bachalor)19-6	2020	1999	21
137	Female	Nursing (bachalor)19-6	2020	2000	20
138	Female	Nursing (bachalor)19-6	2020	1996	24
139	Female	Nursing (bachalor)19-6	2020	1999	21
140	Male	Nursing (bachalor)19-6	2020	2000	20
141	Female	Nursing (bachalor)19-6	2020	2001	19
142	Female	Nursing (bachalor)19-6	2020	1999	21
143	Male	Nursing (bachalor)19-6	2020	1999	21
144	Female	Nursing (bachalor)19-7	2020	2001	19
145	Female	Nursing (bachalor)19-7	2020	2001	19
146	Female	Nursing (bachalor)19-7	2020	2002	18
147	Female	Nursing (bachalor)19-7	2020	2001	19
148	Female	Nursing (bachalor)19-7	2020	2000	20
149	Female	Nursing (bachalor)19-7	2020	1995	25
150	Male	Nursing (bachalor)19-7	2020	1998	22
151	Female	Nursing (bachalor)19-7	2020	1999	21
152	Male	Nursing (bachalor)19-7	2020	2000	20
153	Female	Nursing (bachalor)19-7	2020	2001	19
154	Female	Nursing (bachalor)19-7	2020	1999	21
155	Female	Nursing (bachalor)19-7	2020	2000	20
156	Female	Nursing (bachalor)19-7	2020	2000	20
157	Female	Nursing (bachalor)19-7	2020	1999	21
158	Female	Nursing (bachalor)19-7	2020	2001	19
159	Female	Nursing (bachalor)19-7	2020	2000	20
160	Female	Nursing (bachalor)19-7	2020	2000	20
161	Female	Nursing (bachalor)19-7	2020	1999	21
162	Female	Nursing (bachalor)19-7	2020	2001	19
163	Female	Nursing (bachalor)19-7	2020	2000	20

164	Male	Nursing (bachalor)19-7	2020	2000	20
165	Female	Nursing (bachalor)19-7	2020	2001	19
166	Female	Nursing (bachalor)19-7	2020	1997	23
167	Male	Nursing (bachalor)19-7	2020	1998	22
168	Female	Nursing (bachalor)19-7	2020	2000	20
169	Female	Nursing (bachalor)19-7	2020	2000	20
170	Male	Nursing (bachalor)19-7	2020	2000	20
171	Female	Nursing (bachalor)19-7	2020	1996	24
172	Female	Nursing (bachalor)19-7	2020	2000	20
173	Female	Nursing (bachalor)19-7	2020	2002	18
174	Male	Nursing (bachalor)19-7	2020	2001	19
175	Female	Nursing (bachalor)19-7	2020	1999	21
176	Male	Nursing (bachalor)19-7	2020	1999	21
177	Female	Nursing (bachalor)19-7	2020	2001	19
178	Female	Nursing (bachalor)19-7	2020	2001	19
179	Female	Nursing (bachalor)19-7	2020	2001	19
180	Female	Nursing (bachalor)19-7	2020	1999	21

Teaching Year	Chinese Classic Literature	English	Chinese History	Computer Science	Physiology
Pre-COVID-19	87.0	60.0	81.0	67.0	72.0
Pre-COVID-19	81.0	73.0	74.0	71.0	79.0
Pre-COVID-19	72.0	60.0	76.0	75.0	75.0
Pre-COVID-19	85.0	79.0	85.0	76.0	80.0
Pre-COVID-19	82.0	61.0	82.0	63.8	80.0
Pre-COVID-19	91.0	63.0	87.0	80.0	92.0
Pre-COVID-19	92.0	77.0	87.0	86.0	74.0
Pre-COVID-19	81.0	66.0	82.0	68.0	82.0
Pre-COVID-19	73.0	85.0	79.0	76.0	66.0
Pre-COVID-19	81.0	71.0	85.0	87.0	70.0
Pre-COVID-19	77.0	70.0	80.0	77.0	79.0
Pre-COVID-19	85.0	87.0	77.0	72.0	74.0
Pre-COVID-19	88.0	76.0	89.0	86.0	68.0
Pre-COVID-19	81.0	61.0	79.0	81.0	61.0
Pre-COVID-19	90.0	78.0	87.0	91.0	80.0
Pre-COVID-19	90.0	78.0	86.0	76.0	86.0
Pre-COVID-19	91.0	75.0	83.0	89.0	87.0
Pre-COVID-19	85.0	71.0	83.0	81.0	67.0
Pre-COVID-19	85.0	68.0	85.0	78.0	72.0
Pre-COVID-19	82.0	82.0	77.0	74.0	67.0
Pre-COVID-19	70.0	67.0	81.0	82.0	65.0
Pre-COVID-19	82.0	66.0	80.0	62.9	82.0
Pre-COVID-19	86.0	77.0	88.0	95.0	79.0
Pre-COVID-19	77.0	60.0	82.0	81.0	72.0
Pre-COVID-19	79.0	68.0	79.0	70.0	65.0
Pre-COVID-19	91.0	67.0	84.0	73.0	71.0
Pre-COVID-19	91.0	63.0	74.0	83.0	62.0
Pre-COVID-19	80.0	88.0	86.0	71.0	76.0
Pre-COVID-19	80.0	73.0	87.0	84.0	69.0
Pre-COVID-19	73.0	79.0	84.0	73.0	63.0
Pre-COVID-19	86.0	60.0	82.0	65.0	60.0
Pre-COVID-19	85.0	64.0	88.0	83.0	74.0
Pre-COVID-19	82.0	78.0	88.0	82.0	64.0
Pre-COVID-19	80.0	62.0	91.0	78.0	81.0
Pre-COVID-19	76.0	60.0	83.0	76.0	62.0
Pre-COVID-19	74.0	85.0	83.0	73.0	74.0
Pre-COVID-19	87.0	72.0	88.0	86.0	74.0
Pre-COVID-19	81.0	64.0	85.0	81.0	75.0
Pre-COVID-19	75.0	86.0	78.0	65.0	65.0
Pre-COVID-19	74.0	69.0	80.0	73.0	78.0
Pre-COVID-19	84.0	75.0	86.0	85.0	65.0
Pre-COVID-19	81.0	74.0	82.0	65.0	74.0
Pre-COVID-19	74.0	79.0	86.0	81.0	74.0

Pre-COVID-19	79.0	66.0	76.0	76.0	63.0
Pre-COVID-19	77.0	75.0	75.0	93.0	68.0
Pre-COVID-19	88.0	68.0	69.0	78.0	75.0
Pre-COVID-19	79.0	60.0	62.0	74.0	82.0
Pre-COVID-19	83.0	87.0	64.0	80.0	76.0
Pre-COVID-19	89.0	69.0	85.0	83.0	60.0
Pre-COVID-19	89.0	71.0	74.0	82.0	72.0
Pre-COVID-19	84.0	61.0	77.0	80.0	64.0
Pre-COVID-19	85.0	71.0	81.0	93.0	65.0
Pre-COVID-19	72.0	80.0	83.0	72.0	65.0
Pre-COVID-19	87.0	70.0	88.0	93.0	87.0
Pre-COVID-19	72.0	60.0	76.0	88.0	68.0
Pre-COVID-19	79.0	62.0	87.0	92.0	88.0
Pre-COVID-19	83.0	73.0	74.0	82.0	83.0
Pre-COVID-19	84.0	75.0	84.0	92.0	67.0
Pre-COVID-19	78.0	73.0	74.0	91.0	66.0
Pre-COVID-19	82.0	60.0	82.0	89.0	69.0
Pre-COVID-19	72.0	84.0	85.0	82.0	73.0
Pre-COVID-19	83.0	66.0	85.0	93.0	68.0
Pre-COVID-19	76.0	63.0	85.0	82.0	74.0
Pre-COVID-19	82.0	78.0	85.0	86.0	72.0
Pre-COVID-19	87.0	62.0	86.0	77.0	75.0
Pre-COVID-19	78.0	84.0	81.0	85.0	63.0
Pre-COVID-19	78.0	60.0	86.0	85.0	79.0
Pre-COVID-19	86.0	80.0	82.0	95.0	65.0
Pre-COVID-19	78.0	60.0	76.0	90.0	61.0
Pre-COVID-19	77.0	60.0	78.0	92.0	66.0
Pre-COVID-19	83.0	84.0	81.0	83.0	61.0
Pre-COVID-19	75.0	80.0	71.0	86.0	66.0
Pre-COVID-19	#NULL!	82.0	75.0	92.0	64.0
Pre-COVID-19	74.0	60.0	76.0	84.0	61.0
Pre-COVID-19	82.0	60.0	77.0	87.0	64.0
Pre-COVID-19	80.0	61.0	83.0	79.0	76.0
Pre-COVID-19	85.0	64.0	90.0	89.0	84.0
Pre-COVID-19	79.0	71.0	88.0	92.0	73.0
Pre-COVID-19	79.0	76.0	85.0	90.0	72.0
Pre-COVID-19	79.0	65.0	82.0	95.0	63.0
Pre-COVID-19	87.0	75.0	82.0	96.0	65.0
Pre-COVID-19	81.0	72.0	87.0	74.0	70.0
Pre-COVID-19	80.0	61.0	74.0	82.0	68.0
Pre-COVID-19	70.0	61.0	77.0	93.0	80.0
Pre-COVID-19	83.0	71.0	87.0	91.0	68.0
Pre-COVID-19	77.0	61.0	80.0	72.0	67.0
Pre-COVID-19	78.0	67.0	89.0	90.0	70.0
Pre-COVID-19	75.0	72.0	91.0	92.0	73.0

Pre-COVID-19	80.0	67.0	83.0	78.0	64.0
Pre-COVID-19	77.0	65.0	79.0	85.0	65.0
Pre-COVID-19	82.0	79.0	82.0	93.0	73.0
Pre-COVID-19	75.0	63.0	85.0	74.0	60.0
Pre-COVID-19	82.0	66.0	81.0	82.0	71.0
Pre-COVID-19	79.0	65.0	88.0	86.0	75.0
Pre-COVID-19	79.0	75.0	80.0	86.0	79.0
Pre-COVID-19	73.0	78.0	84.0	90.0	66.0
Pre-COVID-19	75.0	63.0	85.0	93.0	60.0
Pre-COVID-19	72.0	60.0	77.0	85.0	62.0
Pre-COVID-19	74.0	63.0	90.0	84.0	65.0
Pre-COVID-19	86.0	65.0	81.0	66.0	68.0
Pre-COVID-19	80.0	69.0	79.0	74.0	69.0
Pre-COVID-19	79.0	63.0	80.0	93.0	75.0
Pre-COVID-19	79.0	62.0	77.0	88.0	60.0
Pre-COVID-19	69.0	66.0	80.0	84.0	79.0
Pre-COVID-19	75.0	75.0	84.0	89.0	75.0
Pre-COVID-19	68.0	62.0	68.0	82.0	68.0
Pre-COVID-19	84.0	65.0	83.0	92.0	70.0
Pre-COVID-19	74.0	64.0	80.0	86.0	70.0
Pre-COVID-19	82.0	65.0	82.0	87.0	65.0
Pre-COVID-19	81.0	75.0	80.0	94.0	73.0
Pre-COVID-19	74.0	60.0	82.0	73.0	69.0
Pre-COVID-19	88.0	73.0	82.0	96.0	60.0
Pre-COVID-19	81.0	72.0	83.0	92.0	65.0
Pre-COVID-19	84.0	83.0	73.0	95.0	74.0
Pre-COVID-19	78.0	73.0	81.0	88.0	77.0
Pre-COVID-19	64.0	61.0	79.0	64.0	71.0
Pre-COVID-19	83.0	75.0	78.0	91.0	61.0
Pre-COVID-19	68.0	69.0	75.0	79.0	62.0
Pre-COVID-19	86.0	80.0	89.0	93.0	83.0
Pre-COVID-19	84.0	60.0	74.0	92.0	66.0
Pre-COVID-19	74.0	62.0	74.0	95.0	66.0
Pre-COVID-19	75.0	68.0	79.0	75.0	63.0
Pre-COVID-19	70.0	62.0	75.0	86.0	75.0
Pre-COVID-19	81.0	68.0	80.0	90.0	73.0
Pre-COVID-19	75.0	71.0	73.0	82.0	66.0
Pre-COVID-19	79.0	70.0	85.0	86.0	80.0
Pre-COVID-19	80.0	77.0	75.0	60.0	77.0
Pre-COVID-19	73.0	66.5	84.0	89.0	89.0
Pre-COVID-19	74.0	73.0	82.0	78.0	73.0
Pre-COVID-19	77.0	63.0	84.0	85.0	72.0
Pre-COVID-19	76.0	61.0	76.0	90.0	72.0
Pre-COVID-19	83.0	64.0	80.0	91.0	73.0
Pre-COVID-19	75.0	78.0	79.0	90.0	79.0

Pre-COVID-19	84.0	80.0	85.0	80.0	77.0
Pre-COVID-19	81.0	76.0	78.0	82.0	74.0
Pre-COVID-19	81.0	66.0	81.0	81.0	78.0
Pre-COVID-19	84.0	63.0	77.0	80.0	72.0
Pre-COVID-19	75.0	68.0	80.0	79.0	86.0
Pre-COVID-19	86.0	72.0	83.0	76.0	67.0
Pre-COVID-19	70.0	65.5	79.0	70.0	79.0
Pre-COVID-19	80.0	66.0	83.0	91.0	74.0
Pre-COVID-19	82.0	74.0	77.0	90.0	82.0
Pre-COVID-19	83.0	78.0	85.0	91.0	75.0
Pre-COVID-19	78.0	60.0	79.0	76.0	68.0
Pre-COVID-19	85.0	79.0	80.0	94.0	78.0
Pre-COVID-19	78.0	66.0	82.0	88.0	74.0
Pre-COVID-19	89.0	60.0	72.0	87.0	60.0
Pre-COVID-19	81.0	80.0	80.0	93.0	83.0
Pre-COVID-19	77.0	62.0	81.0	82.0	82.0
Pre-COVID-19	88.0	60.0	80.0	84.0	65.0
Pre-COVID-19	82.0	66.0	81.0	88.0	65.0
Pre-COVID-19	83.0	79.0	83.0	89.0	78.0
Pre-COVID-19	93.0	65.0	83.0	69.0	76.0
Pre-COVID-19	81.0	80.0	80.0	88.0	77.0
Pre-COVID-19	84.0	61.0	80.0	90.0	70.0
Pre-COVID-19	76.0	81.0	81.0	83.0	74.0
Pre-COVID-19	75.0	60.0	83.0	74.0	70.0
Pre-COVID-19	72.0	60.0	85.0	84.0	89.0
Pre-COVID-19	89.0	65.0	79.0	89.0	60.0
Pre-COVID-19	81.0	78.0	81.0	74.0	68.0
Pre-COVID-19	76.0	85.0	84.0	82.0	76.0
Pre-COVID-19	93.0	60.0	82.0	82.0	85.0
Pre-COVID-19	90.0	60.0	78.0	76.0	79.0
Pre-COVID-19	78.0	60.0	82.0	92.0	79.0
Pre-COVID-19	81.0	73.0	79.0	92.0	63.0
Pre-COVID-19	75.0	77.0	79.0	75.0	60.0
Pre-COVID-19	78.0	70.0	75.0	86.0	70.0
Pre-COVID-19	85.0	67.0	82.0	96.0	70.0
Pre-COVID-19	88.0	60.0	79.0	67.0	67.0
Pre-COVID-19	90.0	82.0	85.0	87.0	79.0
Pre-COVID-19	74.0	78.0	80.0	78.0	61.0
Pre-COVID-19	78.0	78.0	82.0	89.0	81.0
Pre-COVID-19	80.0	60.0	80.0	86.0	61.0
Pre-COVID-19	76.0	62.0	86.0	83.0	75.0
Pre-COVID-19	70.0	68.0	87.0	79.0	82.0
Pre-COVID-19	82.0	69.0	76.0	94.0	61.0
Pre-COVID-19	77.0	72.0	86.0	82.0	80.0
Pre-COVID-19	62.0	63.0	75.0	80.0	75.0

Pre-COVID-19	81.0	68.5	89.0	87.0	81.0
Pre-COVID-19	85.0	66.0	85.0	93.0	72.0
Pre-COVID-19	78.0	72.0	83.0	94.0	64.0
Pre-COVID-19	72.0	73.0	84.0	89.0	72.0
Pre-COVID-19	90.0	72.0	86.0	80.0	75.0
Pre-COVID-19	82.0	70.0	89.0	79.0	77.0
Pre-COVID-19	75.0	69.0	86.0	90.0	61.0
Pre-COVID-19	79.0	66.0	86.0	93.0	69.0
Pre-COVID-19	89.0	68.0	87.0	71.0	61.0
Pre-COVID-19	79.0	64.0	83.0	88.0	68.0
Pre-COVID-19	79.0	81.0	87.0	92.0	61.0
Pre-COVID-19	85.0	65.0	84.0	87.0	61.0
Pre-COVID-19	84.0	68.0	76.0	88.0	84.0
Pre-COVID-19	81.0	61.0	78.0	88.0	75.0
Pre-COVID-19	83.0	66.0	76.0	95.0	73.0
Pre-COVID-19	75.0	68.0	77.0	87.0	60.0
Pre-COVID-19	84.0	67.0	79.0	85.0	73.0
COVID-19	92.0	62.0	81.0	88.0	66.0
COVID-19	93.0	74.0	87.0	93.0	75.0
COVID-19	94.0	68.0	88.0	95.0	77.0
COVID-19	94.0	75.0	88.0	92.0	84.0
COVID-19	92.0	63.0	79.0	92.0	69.0
COVID-19	94.0	89.0	87.0	88.0	69.0
COVID-19	93.0	81.0	86.0	94.0	76.0
COVID-19	94.0	79.0	88.0	87.0	71.0
COVID-19	86.0	60.0	64.0	69.0	49.0
COVID-19	95.0	84.0	89.0	87.0	74.0
COVID-19	94.0	74.0	88.0	87.0	64.0
COVID-19	93.0	78.0	84.0	78.0	69.0
COVID-19	93.0	76.0	88.0	92.0	74.0
COVID-19	94.0	75.0	88.0	92.0	63.0
COVID-19	92.0	79.0	84.0	85.0	69.0
COVID-19	94.0	79.0	88.0	89.0	67.0
COVID-19	93.0	80.0	88.0	90.0	56.0
COVID-19	90.0	77.0	84.0	90.0	70.0
COVID-19	93.0	81.0	87.0	94.0	73.0
COVID-19	96.0	87.0	88.0	96.0	80.0
COVID-19	94.0	75.0	87.0	93.0	76.0
COVID-19	95.0	65.0	87.0	83.0	72.0
COVID-19	95.0	81.0	86.0	90.0	76.0
COVID-19	91.0	81.0	87.0	72.0	73.0
COVID-19	92.0	69.0	81.0	84.0	70.0
COVID-19	96.0	78.0	89.0	92.0	76.0
COVID-19	91.0	69.0	85.0	72.0	54.0
COVID-19	89.0	82.0	76.0	64.0	62.0

COVID-19	91.0	80.0	88.0	83.0	69.0
COVID-19	92.0	88.0	83.0	75.0	64.0
COVID-19	94.0	82.0	90.0	91.0	83.0
COVID-19	89.0	76.0	88.0	82.0	74.0
COVID-19	78.9	60.0	72.0	82.0	60.0
COVID-19	92.0	78.0	87.0	88.0	75.0
COVID-19	93.0	87.0	85.0	74.0	73.0
COVID-19	95.0	88.0	89.0	87.0	74.0
COVID-19	90.0	74.0	86.0	79.0	79.0
COVID-19	96.0	82.0	87.0	73.0	75.0
COVID-19	95.0	85.0	67.0	93.0	78.0
COVID-19	92.0	80.0	86.0	90.0	65.0
COVID-19	94.0	73.0	85.0	81.0	77.0
COVID-19	94.0	84.0	87.0	88.0	76.0
COVID-19	93.0	87.0	88.0	83.0	75.0
COVID-19	96.0	86.0	87.0	91.0	80.0
COVID-19	93.0	85.0	84.0	83.0	61.0
COVID-19	94.0	89.0	86.0	84.0	75.0
COVID-19	95.0	79.0	87.0	93.0	79.0
COVID-19	94.0	88.0	89.0	96.0	80.0
COVID-19	91.0	75.0	83.0	74.0	67.0
COVID-19	91.0	#NULL!	89.0	94.0	78.0
COVID-19	84.0	73.0	85.0	92.0	61.0
COVID-19	92.0	88.0	87.0	87.0	72.0
COVID-19	94.0	83.0	85.0	80.0	70.0
COVID-19	96.0	85.0	88.0	95.0	74.0
COVID-19	94.0	85.0	87.0	93.0	81.0
COVID-19	92.0	86.0	88.0	77.0	80.0
COVID-19	94.0	88.0	87.0	82.0	67.7
COVID-19	93.0	76.0	88.0	92.0	76.0
COVID-19	92.0	79.0	84.0	74.0	68.0
COVID-19	94.0	89.0	88.0	92.0	70.0
COVID-19	95.0	70.0	85.0	67.0	57.0
COVID-19	85.0	78.0	86.0	79.0	76.0
COVID-19	94.0	87.0	88.0	93.0	79.0
COVID-19	94.0	84.0	88.0	94.0	74.0
COVID-19	94.0	82.0	86.0	81.0	69.0
COVID-19	83.0	77.0	86.0	74.0	68.0
COVID-19	93.0	84.0	86.0	83.0	73.0
COVID-19	95.0	81.0	67.0	88.0	69.0
COVID-19	96.0	81.0	86.0	82.0	71.0
COVID-19	95.0	88.0	85.0	85.0	77.0
COVID-19	93.0	88.0	88.0	86.0	72.0
COVID-19	94.0	90.0	88.0	90.0	78.0
COVID-19	89.0	73.0	89.0	69.0	62.0

COVID-19	93.0	81.0	90.0	89.0	76.0
COVID-19	96.0	85.0	91.0	90.0	70.0
COVID-19	90.0	64.0	83.0	73.0	76.0
COVID-19	87.0	62.0	92.0	87.0	62.0
COVID-19	96.0	88.0	94.0	94.0	77.0
COVID-19	83.0	76.0	90.0	70.0	66.0
COVID-19	92.0	80.0	88.0	93.0	81.0
COVID-19	86.0	64.0	82.0	80.0	66.0
COVID-19	94.0	81.0	91.0	91.0	69.0
COVID-19	91.0	70.0	89.0	76.0	73.0
COVID-19	91.0	82.0	90.0	80.0	68.0
COVID-19	81.0	65.0	91.0	76.0	61.0
COVID-19	68.0	74.0	89.0	77.0	56.0
COVID-19	66.0	67.0	81.0	90.0	47.0
COVID-19	83.0	81.0	90.0	75.0	70.0
COVID-19	96.0	99.0	93.0	73.0	81.0
COVID-19	77.0	63.0	85.0	74.0	63.0
COVID-19	95.0	88.0	93.0	90.0	76.0
COVID-19	72.0	63.0	85.0	72.0	66.0
COVID-19	93.0	88.0	93.0	96.0	76.0
COVID-19	90.0	79.0	91.0	79.0	78.0
COVID-19	94.0	77.0	91.0	92.0	80.0
COVID-19	86.0	69.0	91.0	92.0	83.0
COVID-19	75.0	81.0	89.0	67.0	76.0
COVID-19	82.0	66.0	89.0	62.0	67.0
COVID-19	89.0	77.0	86.0	85.0	66.0
COVID-19	91.0	62.0	90.0	67.0	66.0
COVID-19	81.0	81.0	83.0	71.0	66.0
COVID-19	82.0	62.0	89.0	82.0	61.0
COVID-19	81.0	74.0	85.0	88.0	67.0
COVID-19	87.0	74.0	89.0	74.0	66.0
COVID-19	89.0	79.0	90.0	29.0	74.0
COVID-19	93.0	83.0	91.0	93.0	70.0
COVID-19	69.0	72.0	85.0	80.0	75.0
COVID-19	80.0	75.0	89.0	73.0	70.0
COVID-19	80.0	55.0	89.0	80.0	57.0
COVID-19	77.0	82.0	90.0	88.0	70.0
COVID-19	96.0	81.0	91.0	87.0	78.0
COVID-19	94.0	93.0	92.0	82.0	61.0
COVID-19	84.0	77.0	86.0	85.0	62.0
COVID-19	89.0	74.0	90.0	84.0	70.0
COVID-19	83.0	73.0	87.0	62.0	66.0
COVID-19	95.0	82.0	89.0	93.0	83.0
COVID-19	86.0	82.0	89.0	82.0	65.0
COVID-19	90.0	70.0	90.0	95.0	78.0

COVID-19	94.0	84.0	91.0	92.0	80.0
COVID-19	80.0	85.0	91.0	91.0	67.0
COVID-19	68.0	71.0	84.0	90.0	79.0
COVID-19	78.0	85.0	90.0	85.0	65.0
COVID-19	93.0	81.0	88.0	95.0	73.0
COVID-19	94.0	76.0	90.0	88.0	68.0
COVID-19	77.0	83.0	86.0	93.0	73.0
COVID-19	63.0	64.0	81.0	72.0	62.0
COVID-19	81.0	84.0	87.0	71.0	63.0
COVID-19	95.0	76.0	89.0	78.0	68.0
COVID-19	79.0	69.0	84.0	62.0	67.0
COVID-19	89.0	86.0	88.0	95.0	78.0
COVID-19	73.0	74.0	89.0	88.0	73.0
COVID-19	68.0	72.0	86.0	96.0	61.0
COVID-19	75.0	74.0	90.0	71.0	74.0
COVID-19	95.0	83.0	91.0	93.0	76.0
COVID-19	71.0	68.0	68.0	61.0	66.0
COVID-19	76.0	81.0	85.0	78.0	71.0
COVID-19	97.0	82.0	90.0	93.0	86.0
COVID-19	93.0	84.0	90.0	95.0	72.0
COVID-19	93.0	77.0	90.0	93.0	73.0
COVID-19	65.0	60.0	63.0	78.0	50.0
COVID-19	97.0	74.0	91.0	85.0	79.0
COVID-19	96.0	63.0	88.0	77.0	64.0
COVID-19	74.0	62.0	64.0	89.0	71.0
COVID-19	83.0	85.0	91.0	76.0	73.0
COVID-19	93.0	84.0	90.0	81.0	76.0
COVID-19	96.0	79.0	91.0	84.0	76.0
COVID-19	95.0	86.0	90.0	79.0	80.0
COVID-19	82.0	75.0	87.0	64.0	69.0
COVID-19	90.0	78.0	88.0	89.0	84.0
COVID-19	93.0	80.0	89.0	83.0	81.0
COVID-19	93.0	68.0	90.0	64.0	73.0
COVID-19	87.0	86.0	89.0	84.0	75.0
COVID-19	93.0	75.0	87.0	79.0	77.0
COVID-19	91.0	62.0	87.0	66.0	75.0
COVID-19	85.0	68.0	86.0	85.0	80.0
COVID-19	69.0	82.0	76.0	87.0	74.0
COVID-19	81.0	62.0	86.0	85.0	80.0
COVID-19	94.0	85.0	90.0	92.0	67.0
COVID-19	77.0	66.0	88.0	98.0	71.0
COVID-19	93.0	82.0	90.0	79.0	76.0
COVID-19	95.0	85.0	92.0	78.0	77.0
COVID-19	95.0	70.0	92.0	82.0	83.0
COVID-19	92.0	80.0	89.0	85.0	82.0

COVID-19	64.0	71.0	82.0	66.0	70.0
COVID-19	78.0	82.0	88.0	79.0	78.0
COVID-19	84.0	78.0	90.0	89.0	72.0
COVID-19	70.0	83.0	76.0	81.0	66.0
COVID-19	90.0	61.0	86.0	65.0	81.0
COVID-19	63.0	71.0	91.0	92.0	79.0
COVID-19	82.0	74.0	87.0	93.0	75.0
COVID-19	76.0	77.0	91.0	94.0	78.0
COVID-19	93.0	75.0	92.0	92.0	64.0
COVID-19	90.0	70.0	88.0	68.0	78.0
COVID-19	76.0	89.0	88.0	79.0	80.0
COVID-19	84.0	86.0	91.0	80.0	76.0
COVID-19	88.0	83.0	90.0	88.0	75.0
COVID-19	87.0	71.0	90.0	85.0	79.0
COVID-19	85.0	82.0	94.0	85.0	85.0
COVID-19	93.0	67.0	87.0	88.0	69.0
COVID-19	83.0	74.0	90.0	76.0	71.0

Biochemistry	Patho immunology	Physiological Practice	TCM Nursing	Combined Nursing
84.0	82.0	85.0	87.0	73.0
85.0	86.0	81.0	83.0	90.0
70.0	71.0	82.0	73.0	63.0
82.0	85.0	89.0	80.0	74.0
80.0	83.0	84.0	76.0	62.0
92.0	89.0	87.0	95.0	83.0
93.0	88.0	87.0	85.0	79.0
85.0	77.0	85.0	85.0	86.0
78.0	82.0	81.0	74.0	70.0
73.0	68.0	77.0	76.0	74.0
75.0	60.5	80.0	78.0	76.0
74.0	74.0	79.0	76.0	74.0
92.0	89.0	84.0	87.0	87.0
71.0	67.0	86.0	79.0	69.0
86.0	88.0	84.0	83.0	76.0
89.0	85.0	85.0	91.0	83.0
86.0	81.0	79.0	76.0	86.0
70.0	72.0	78.0	79.0	74.0
83.0	84.0	81.0	81.0	60.0
73.0	64.0	79.0	75.0	68.0
64.0	78.0	84.0	#NULL!	83.0
65.0	68.0	82.0	72.0	68.0
89.0	80.0	84.0	77.0	61.0
68.0	74.0	85.0	80.0	66.0
71.0	65.0	79.0	78.0	65.0
82.0	60.0	82.0	86.0	67.0
83.0	68.0	75.0	82.0	68.0
79.0	74.0	87.0	82.0	85.0
76.0	68.0	81.0	85.0	80.0
67.0	69.0	79.0	82.0	60.0
69.0	62.0	82.0	73.0	71.0
77.0	76.0	84.0	80.0	74.0
74.0	81.0	88.0	97.0	91.0
87.0	82.0	88.0	80.0	73.0
80.0	74.0	75.0	86.0	66.0
79.0	73.0	85.0	79.0	69.0
84.0	71.0	83.0	81.0	90.0
82.0	73.0	86.0	78.0	67.0
60.0	64.0	72.0	76.0	65.0
78.0	60.5	82.0	73.0	73.0
74.0	63.0	85.0	86.0	78.0
72.0	65.0	86.0	79.0	75.0
78.0	91.0	93.0	95.0	86.0

63.0	66.0	83.0	74.0	69.0
78.0	75.0	82.0	74.0	71.0
70.0	66.0	80.0	78.0	67.0
82.0	64.0	81.0	77.0	61.0
73.0	64.0	76.0	68.0	74.0
68.0	71.0	76.0	83.0	81.0
75.0	65.0	82.0	81.0	68.0
72.0	75.0	80.0	79.0	73.0
70.0	75.0	88.0	77.0	78.0
71.0	66.0	87.0	75.0	68.0
88.0	85.0	88.0	89.0	91.0
68.0	75.0	88.0	69.0	60.0
90.0	85.0	87.0	96.0	84.0
85.0	83.0	85.0	77.0	66.0
74.0	79.0	90.0	81.0	77.0
82.0	71.0	87.0	76.0	70.0
68.0	77.0	84.0	83.0	66.0
75.0	63.0	80.0	70.0	60.0
71.0	71.0	85.0	80.0	75.0
81.0	73.0	82.0	81.0	69.0
66.0	71.0	86.0	80.0	66.0
69.0	81.0	89.0	86.0	83.0
70.0	75.0	84.0	79.0	78.0
85.0	78.0	83.0	80.0	77.0
64.0	60.0	84.0	75.0	70.0
76.0	66.0	75.0	78.0	62.0
61.0	60.0	74.0	66.0	71.0
76.0	73.0	81.0	74.0	61.0
69.0	76.0	81.0	77.0	65.0
73.0	72.0	75.0	72.0	75.0
81.0	72.0	75.0	80.0	68.0
86.0	82.0	79.0	81.0	62.0
82.0	78.0	81.0	84.0	80.0
81.0	88.0	85.0	90.0	81.0
76.0	81.0	80.0	84.0	79.0
87.0	79.0	82.0	72.0	72.0
75.0	77.0	79.0	83.0	79.0
76.0	79.0	81.0	83.0	74.0
86.0	80.0	83.0	76.0	84.0
80.0	70.0	84.0	76.0	67.0
93.0	83.0	79.0	78.0	71.0
70.0	77.0	82.0	89.0	75.0
72.0	77.0	82.0	87.0	68.0
71.0	73.0	83.0	83.0	74.0
80.0	83.0	86.0	83.0	72.0

76.0	79.0	81.0	82.0	79.0
73.0	76.0	84.0	79.0	69.0
78.0	83.0	82.0	86.0	78.0
79.0	74.0	80.0	76.0	73.0
63.0	74.0	81.0	73.0	64.0
85.0	78.0	79.0	81.0	69.0
81.0	80.0	75.0	81.0	73.0
66.0	74.0	81.0	68.0	63.0
78.0	68.0	76.0	74.0	72.0
66.0	77.0	79.0	71.0	70.0
67.0	77.0	76.0	73.0	71.0
75.0	70.0	80.0	84.0	70.0
70.0	69.0	78.0	81.0	75.0
85.0	74.0	84.0	85.0	71.0
60.0	73.0	79.0	74.0	75.0
83.0	72.0	78.0	84.0	85.0
86.0	73.0	83.0	84.0	83.0
70.0	66.0	79.0	80.0	75.0
80.0	75.0	88.0	76.0	80.0
80.0	69.0	80.0	79.0	73.0
78.0	74.0	81.0	81.0	84.0
78.0	73.0	78.0	76.0	81.0
73.0	62.0	82.0	80.0	80.0
69.0	70.0	76.0	86.0	72.0
78.0	69.0	85.0	83.0	61.0
75.0	68.0	80.0	80.0	80.0
82.0	74.0	81.0	70.0	82.0
65.0	70.0	87.0	83.0	80.0
75.0	81.0	81.0	79.0	82.0
64.0	67.0	74.0	76.0	74.0
79.0	75.0	83.0	87.0	79.0
66.0	62.0	81.0	80.0	67.0
79.0	71.0	78.0	80.0	73.0
81.0	65.0	62.0	82.0	74.0
86.0	76.0	79.0	84.0	64.0
78.0	71.0	74.0	81.0	70.0
79.0	62.0	79.0	88.0	73.0
86.0	81.0	88.0	88.0	81.0
73.0	84.0	71.0	85.0	80.0
92.0	84.0	78.0	90.0	88.0
63.0	74.0	89.0	83.0	83.0
83.0	78.0	87.0	87.0	76.0
67.0	77.0	89.0	83.0	72.0
79.0	86.0	87.0	89.0	85.0
83.0	77.0	80.0	93.0	77.0

73.0	83.0	91.0	94.0	80.0
73.0	74.0	80.0	86.0	74.0
85.0	83.0	78.0	95.0	80.0
61.0	76.0	79.0	88.0	74.0
82.0	82.0	82.0	88.0	89.0
72.0	77.0	88.0	85.0	80.0
63.0	79.0	73.0	74.0	83.0
70.0	79.0	79.0	71.0	73.0
85.0	83.0	89.0	82.0	84.0
77.0	79.0	87.0	93.0	79.0
75.0	79.0	82.0	87.0	66.0
77.0	77.0	78.0	78.0	72.0
77.0	72.0	81.0	78.0	74.0
71.0	73.0	61.0	84.0	69.0
82.0	84.0	75.0	88.0	73.0
80.0	84.0	93.0	93.0	76.0
85.0	74.0	90.0	90.0	71.0
83.0	75.0	80.0	82.0	78.0
83.0	78.0	92.0	91.0	75.0
76.0	78.0	83.0	92.0	79.0
72.0	82.0	88.0	73.0	78.0
82.0	66.0	89.0	92.0	77.0
67.0	74.0	88.0	87.0	75.0
75.0	72.0	85.0	87.0	71.0
89.0	89.0	84.0	93.0	75.0
78.0	74.0	83.0	75.0	74.0
81.0	69.0	83.0	89.0	80.0
84.0	73.0	88.0	78.0	65.0
90.0	85.0	88.0	90.0	80.0
86.0	78.0	88.0	90.0	75.0
87.0	85.0	87.0	86.0	81.0
76.0	76.0	76.0	80.0	74.0
65.0	80.0	72.0	75.0	60.0
76.0	77.0	81.0	90.0	64.0
90.0	76.0	82.0	90.0	74.0
82.0	70.0	86.0	86.0	77.0
79.0	78.0	83.0	92.0	62.0
71.0	60.0	75.0	85.0	76.0
80.0	75.0	88.0	88.0	76.0
72.0	77.0	82.0	85.0	66.0
89.0	75.0	75.0	88.0	72.0
89.0	71.0	83.0	85.0	77.0
72.0	71.0	82.0	87.0	73.0
87.0	80.0	83.0	89.0	79.0
75.0	72.0	83.0	81.0	62.0

83.0	78.0	79.0	82.0	76.0
86.0	76.0	85.0	84.0	87.0
88.0	77.0	87.0	89.0	81.0
86.0	78.0	88.0	91.0	72.0
87.0	72.0	81.0	85.0	78.0
89.0	84.0	85.0	92.0	84.0
87.0	81.0	93.0	75.0	78.0
77.0	76.0	88.0	91.0	67.0
87.0	65.0	84.0	93.0	72.0
85.0	74.0	80.0	89.0	73.0
77.0	65.0	88.0	88.0	86.0
73.0	77.0	79.0	84.0	66.0
83.0	80.0	79.0	84.0	74.0
82.0	79.0	86.0	84.0	78.0
75.0	78.0	72.0	80.0	77.0
79.0	73.0	78.0	81.0	65.0
89.0	80.0	83.0	77.0	70.0
76.0	61.0	88.0	91.0	79.0
65.0	75.0	88.0	80.0	85.0
74.0	78.0	91.0	87.0	74.0
81.0	69.0	89.0	93.0	88.0
68.0	72.0	90.0	94.0	78.0
78.0	79.0	87.0	93.0	83.0
76.0	82.0	91.0	94.0	91.0
71.0	72.0	88.0	91.0	76.0
61.0	69.0	89.0	89.0	82.0
76.0	77.0	92.0	96.0	86.0
64.0	77.0	92.0	96.0	82.0
50.0	72.0	91.0	80.0	91.0
68.0	77.0	90.0	88.0	87.0
71.0	71.0	90.0	95.0	77.0
69.0	65.0	90.0	83.0	88.0
66.0	80.0	89.0	95.0	85.0
58.0	72.0	92.0	87.0	83.0
75.0	79.0	90.0	83.0	74.0
65.0	75.0	88.0	87.0	88.0
73.0	84.0	91.0	93.0	92.0
78.0	67.0	92.0	92.0	80.0
70.0	76.0	85.0	89.0	83.0
69.0	82.0	91.0	89.0	85.0
69.0	80.0	88.0	89.0	86.0
56.0	63.0	89.0	82.0	76.0
68.0	85.0	89.0	94.0	80.0
65.0	69.0	86.0	83.0	76.0
54.0	67.0	86.0	#NULL!	79.0

60.0	79.0	89.0	88.0	95.0
56.0	65.0	88.0	83.0	65.0
90.0	86.0	92.0	87.0	90.0
67.0	78.0	88.0	88.0	89.0
69.0	67.0	90.0	79.4	82.0
73.0	79.0	93.0	82.0	88.0
68.0	86.0	90.0	86.0	89.0
68.0	81.0	86.0	92.0	85.0
78.0	81.0	89.0	82.0	88.0
71.0	89.0	84.0	97.0	90.0
76.0	85.0	83.0	96.0	91.0
65.0	78.0	85.0	91.0	88.0
67.0	86.0	83.0	90.0	86.0
76.0	87.0	84.0	94.0	86.0
66.0	85.0	86.0	82.0	93.0
66.0	88.0	82.0	95.0	88.0
66.0	79.0	85.0	95.0	84.0
76.0	74.0	90.0	83.0	90.0
74.0	80.0	86.0	87.0	84.0
75.0	80.0	87.0	89.0	86.0
64.0	74.0	86.0	83.0	84.0
80.0	79.0	82.0	95.0	90.0
66.0	65.0	81.0	84.0	79.0
68.0	76.0	84.0	82.0	82.0
66.0	77.0	85.0	87.0	81.0
65.0	86.0	82.0	94.0	94.0
78.0	82.0	85.0	92.0	90.0
74.0	83.0	83.0	88.0	90.0
60.0	81.0	82.0	97.0	82.0
75.0	85.0	86.0	97.0	91.0
64.0	79.0	84.0	89.0	83.0
66.0	81.0	87.0	97.0	93.0
50.0	73.0	82.0	78.0	93.0
62.0	75.0	84.0	70.0	75.0
67.0	85.0	84.0	91.0	91.0
73.0	78.0	85.0	96.0	89.0
82.0	72.0	83.0	92.0	86.0
63.0	81.0	84.0	74.0	78.0
70.0	82.0	88.0	87.0	86.0
74.0	88.0	86.0	93.0	88.0
67.0	78.0	84.0	85.0	79.0
70.0	86.0	87.0	83.0	89.0
69.0	88.0	87.0	92.0	86.0
65.0	91.0	87.0	92.0	89.0
66.0	66.0	91.0	79.0	70.0

69.0	77.0	89.0	88.0	81.0
78.0	77.0	91.0	86.0	78.0
76.0	68.0	94.0	85.0	63.0
76.0	79.0	89.0	82.0	83.0
82.0	86.0	92.0	95.0	82.0
68.0	79.0	90.0	90.0	79.0
67.0	76.0	91.0	91.0	85.0
62.0	61.0	91.0	91.0	78.0
71.0	81.0	91.0	91.0	87.0
64.0	72.0	92.0	94.0	87.0
67.0	75.0	91.0	86.0	86.0
69.0	70.0	92.0	91.0	73.0
62.0	80.0	88.0	86.0	73.0
79.0	67.0	89.0	83.0	74.0
68.0	83.0	88.0	82.0	78.0
75.0	94.0	87.0	94.0	93.0
76.0	73.0	88.0	77.0	60.0
82.0	84.0	91.0	95.0	89.0
64.0	72.0	91.0	85.0	76.0
67.0	89.0	89.0	97.0	86.0
66.0	83.0	92.0	75.0	81.0
73.0	90.0	90.0	92.0	89.0
77.0	89.0	91.0	93.0	86.0
68.0	82.0	96.0	88.0	77.0
61.0	65.0	91.0	76.0	67.0
58.0	68.0	92.0	85.0	82.0
65.0	76.0	88.0	79.0	77.0
64.0	80.0	91.0	84.0	80.0
70.0	78.0	90.0	83.0	83.0
64.0	73.0	91.0	85.0	83.0
66.0	83.0	89.0	85.0	86.0
65.0	67.0	86.0	88.0	87.0
66.0	70.0	90.0	88.0	85.0
66.0	64.0	89.0	84.0	74.0
67.0	75.0	91.0	85.0	84.0
49.0	64.0	92.0	71.0	78.0
72.0	91.0	93.0	92.0	87.0
61.0	85.0	93.0	85.0	90.0
67.0	89.0	94.0	88.0	84.0
60.0	70.0	87.0	72.0	88.0
83.0	79.0	87.0	93.0	74.0
65.0	79.0	90.0	90.0	75.0
77.0	85.0	92.0	95.0	87.0
65.0	77.0	91.0	90.0	83.0
78.0	92.0	92.0	86.0	86.0

73.0	82.0	91.0	92.0	86.0
70.0	84.0	91.0	94.0	86.0
68.0	70.0	94.0	89.0	91.0
73.0	82.0	92.0	94.0	71.0
65.0	90.0	91.0	94.0	84.0
66.0	92.0	91.0	92.0	86.0
67.0	77.0	91.0	96.0	80.0
60.0	61.9	91.0	76.0	63.0
65.0	75.0	89.0	79.0	86.0
74.0	89.0	90.0	95.0	80.0
69.0	61.0	90.0	76.0	74.0
76.0	78.0	93.0	86.0	82.0
66.0	78.0	92.0	84.0	83.0
67.0	79.0	90.0	76.0	74.0
72.0	84.0	92.0	97.0	81.0
67.0	92.0	94.0	95.0	85.0
68.0	69.0	90.0	81.0	68.0
63.0	82.0	92.0	85.0	74.0
77.0	93.0	92.0	96.0	86.0
66.0	87.0	90.0	89.0	86.0
69.0	91.0	91.0	85.0	81.0
51.0	60.5	91.0	53.0	76.0
78.0	81.0	89.0	84.0	79.0
66.0	77.0	91.0	79.0	79.0
71.0	81.0	91.0	94.0	87.0
65.0	77.0	88.0	80.0	76.0
70.0	72.0	89.0	91.0	89.0
66.0	89.0	93.0	85.0	83.0
73.0	87.0	89.0	92.0	86.0
66.0	77.0	88.0	78.0	81.0
80.0	91.0	89.0	85.0	89.0
62.0	73.0	91.0	85.0	88.0
69.0	82.0	90.0	82.0	70.0
74.0	88.0	90.0	68.0	83.0
76.0	94.0	91.0	88.0	88.0
79.0	78.0	86.0	87.0	85.0
68.0	80.0	83.0	84.0	81.0
66.0	91.0	88.0	79.0	74.0
72.0	75.0	87.0	81.0	85.0
67.0	90.0	91.0	85.0	84.0
56.0	64.0	88.0	76.0	80.0
68.0	87.0	88.0	88.0	85.0
67.0	88.0	89.0	91.0	90.0
72.0	87.0	90.0	86.0	88.0
76.0	90.0	88.0	89.0	80.0

64.0	68.0	90.0	65.0	75.0
63.0	83.0	87.0	76.0	77.0
65.0	83.0	85.0	77.0	84.0
55.0	65.0	90.0	83.0	78.0
62.0	78.0	85.0	70.0	69.0
76.0	83.0	91.0	72.0	85.0
63.0	91.0	92.0	84.0	88.0
65.0	91.0	92.0	83.0	72.0
74.0	89.0	92.0	81.0	84.0
64.0	81.0	91.0	89.0	79.0
71.0	79.0	85.0	88.0	91.0
72.0	87.0	90.0	89.0	86.0
66.0	67.0	90.0	90.0	87.0
62.0	83.0	89.0	89.0	93.0
70.0	88.0	92.0	82.0	85.0
65.0	85.0	87.0	86.0	81.0
60.0	81.0	89.0	87.0	78.0
