# Supplementary material for: The impact of COVID-19 lockdown on nursing higher education at Chengdu University
Source: PLoS One. 2023 Jun 16;18(6):e0286290. doi: 10.1371/journal.pone.0286290 (PMC10275429; doi:10.1371/journal.pone.0286290)
Supplement: Attachment — Submitted filename: rebuttal V8.docx [file pone.0286290.s002.docx]

PONE-D-22-15585

The impact of COVID-19 lockdown in nursing higher education of Chengdu University

Dr Omar Mohammad Ali Khraisat,

Academic Editor

PLOS ONE

Dear Dr Khraisat,

We appreciate the constructive comments made by the reviewers and our responses are as follows:

Reviewer #1:

The title: *Capital letters are used in the title. The title should be corrected based on the journal's style.*

We have revised according to the Journal Style, it now reads: “The impact of COVID-19 lockdown in nursing higher education of Chengdu University”

Abstract:
*-- Please explain more about the aim and method of the study in the abstract section.*

We have modified the Abstract accordingly, it now reads:

“*Purpose*: To determine the impact of a long-term lockdown on the academic performance of first‑year nursing students *via* online learning, and benefit of online teaching.

*Methods*: The recruitments and academic performance of 1^st^‑year nursing students were assessed between 2019 [prior to, n = 195, (49 men and 146 women)] and 2020 [during COVID-19, n = 180 (38 men and 142 women)]. The independent sample *t* test or Mann‑Whitney was applied for a comparison between these two sets.”

*-- What kind of statistical tests were used?*

To clarify this point, we have added the following sentences in the statistical analysis, it now reads: “The Shapiro-Wilkerson test was utilized to evaluate whether the scores of each curriculum conformed to a normal distribution and to further compare the two sets of data. The quantitative information that adhered to the normal distribution was represented statistically using the mean and standard deviation ($\bar{x}\pm s$) and the independent sample *t* test (homogeneous variance) or *t'* test (uneven variance) was used as a comparison between these two sets. For continuous variables that deviated from the normal distribution, that is, that showed a skewed distribution, this data was expressed using the median and interquartile range [M (*P*25, *P*75)] and the Mann-Whitney U test was applied to test the association between the two groups.” (Materials and Methods: page 6, para 4, lines 197-198).

Introduction
*---You have explained well the importance of complete lockdown in COVID-19, but have not said anything about the importance of proper training of the nursing students.*

To address this point, we have added the following sentences in the Introduction, it now reads: “However, the impact of lockdown in the training of nursing students remains to be explored. The importance of training competent nursing students is well known, who will become specialized registered nurses and play a critical role in the medical practices either in hospitals and/or private practices. On the other hand, incompetent nurses would compromise health care delivery at various different levels of outcomes for the management of patients.” (Introduction, last para, line 103-107).

*--- What is the difference between your study and previous studies have been conducted in this field? Briefly explain in this section.*

To address this point, we have added the following sentences, it now reads: “It has been reported that the mental health has been significantly harmed by the pandemic, e.g. increased stress level, including fear of infection and isolation following long-term lockdown [12] among general populations, and anxiety among medical staff and medical students, perhaps due to excessive media consumption that was linked to mood disorders during the lockdown in China [13, 14].” (Introduction, page 3, para 4, line 104-108).

*--- The limitations of prior research might also be mentioned by the authors as further support for their present investigation.*

To address this point, we have added the following sentences, it now reads: “Online teaching has been introduced previously, demonstrating promising outcomes for medical and/or nursing students. The previous studies are focusing on general population and/or medical staff. However, it remains to be explored what the impact of lockdown is on nursing students. In addition, the online teaching in the previous study is a supplementary approach for face‑to‑face teaching, but not exclusive online teaching as presented in the current study.” (Introduction, page 3, para 2, lines 97-102).

Methods
*---Please write the type of study, sample size, data collection tool and sampling strategy*

*--- Mention the number of samples examined.*

To address this point, we have added the following sentences, it now reads: “This study was an observational retrospective study. The study participants were from 1^st^ year Nursing students in Preclinical School of Chengdu University. All research data including students’ gender, age and academic performance were based on electronic teaching management system of Chengdu University. In the current study, convenience sampling was adopted. There were 195 students in pre-COVID-19 cohort, including 49 men and 146 women. And there were 180 students in COVID-19 cohort, consisting of 38 men and 142 women.” (Materials and Methods, page 5, para 1, line 129-135).

Results:
*---Mention the age range of participants.*

To address this point, we have added the following sentences, it now reads: “The age of pre‑COVID-19 cohort was (20.1 ± 1.3) years, including (19.9 ± 1.2) years for men and (20.2 ± 1.4) years for women. And the age of COVID-19 cohort was (20.1 ± 1.5) years, consisting of (20.6 ± 1.7) years for men and (20.0 ± 1.5) years for women (Table 1). (Results, page 7, para 1, lines 211-214).

Table 1 Characteristics of the students between the pre-COVID-19 cohort and COVID-19 cohort

|  |  | Total Number | pre-COVID-19 cohort (n=195) | COVID-19  cohort (n=180) | *t* / Chi-square test | |
| --- | --- | --- | --- | --- | --- | --- |
|  |  |  |  |  | *t* / *χ^2^* value | *P* value |
| Age (years) |  | 375 | 20.1 ± 1.3 | 20.1 ± 1.5 | 0.015 | 0.988 |
| Age (years) | men | 87 | 19.9 ± 1.2 | 20.6 ± 1.7 | -2.307 | 0.024 |
|  | women | 288 | 20.2 ± 1.4 | 20.0 ± 1.5 | 1.228 | 0.220 |
| Sex n (%) | men | 87 | 49 (25.1) | 38 (21.1) | 0.848 | 0.357 |
|  | women | 288 | 146 (74.9) | 142 (78.9) |  |  |

*---Line 197: Was the method of taking the exam the same in both groups? This should be specified, for example, one group of face-to-face test and another group of online test can have an effect on the average score obtained in the two groups.*

To address this point, we have added the following sentences, it now reads: “Furthermore, the two cohorts carried out their final tests in the same way, that is offline tests, when the students returned to the university after the outbreak.” (Materials and Methods, page 6, para 3 lines 193-195).

*--- It should be specified that the performance score of the students in each course is calculated as an average score? If the average score is calculated, the average score of each course in two groups should be given in a table.*

We have modified accordingly, and presented the data in tables (Table 2-7).

*---Reporting the results in the form of a table to compare two groups based on demographic variables makes the work more beautiful and understandable.*

We have modified accordingly, and showed data in tables (Table 2-7).”

*--- It is better mention the possible score (range) for each subjects (Chinese Classic Literature, English, Computer, Chinese History and Combined Training,....) so easier to readers interpret the results.*

We have modified accordingly, and showed data in tables (Tables 2-7).

Table 2 Academic achievements comparison of basic courses between the pre-COVID-19 cohort and COVID-19 cohort ($\bar{x}\pm s$)

| Courses | pre-COVID-19 cohort (n=195) | COVID-19 cohort (n=180) | mean difference,  95% CI | *t* value | *P* value |
| --- | --- | --- | --- | --- | --- |
| Chinese Classic Literature | 80.21 ± 5.73 | 88.11 ± 8.23 | -7.90 (-9.36- -6.45) | -10.698 | <0.001 |
| English | 69.44 ± 7.55 | 77.39 ± 8.17 | -7.95 (-9.55- -6.35) | -9.780 | <0.001 |
| Chinese History | 81.25 ± 4.84 | 86.86 ± 5.16 | -5.60 (-6.62- -4.59) | -10.851 | <0.001 |
| Computer Science | 83.43 ± 8.12 | 83.19 ± 9.80 | 0.25 (-1.58- 2.07) | 0.265 | 0.791 |

Table 3 Academic achievements comparison of basic courses between the pre-COVID-19 cohort and COVID-19 cohort based on sex stratification ($\bar{x}\pm s$)

| Courses | Group | pre-COVID-19 cohort (n=195) | COVID-19 cohort (n=180) | mean difference, 95% CI | *t* value | *P* value |
| --- | --- | --- | --- | --- | --- | --- |
| Chinese Classic Literature | men | 80.40 ± 5.67 | 86.05 ± 9.12 | -5.66 (-9.04- -2.27) | -3.346 | 0.001 |
|  | women | 80.14 ± 5.77 | 88.66 ± 7.92 | -8.52 (-10.13- -6.91) | -10.405 | <0.001 |
| English | men | 69.53 ± 7.70 | 78.13 ± 8.18 | -8.60 (-12.00- -5.20) | -5.028 | <0.001 |
|  | women | 69.41 ± 7.52 | 77.19 ± 8.18 | -7.78 (-9.60- -5.95） | -8.387 | <0.001 |
| Chinese History | men | 78.31 ± 5.69 | 84.42 ± 7.03 | -6.11 (-8.83- -3.40） | -4.485 | <0.001 |
|  | women | 82.24 ± 4.09 | 87.51 ± 4.34 | -5.27 (-6.24- -4.29） | -10.606 | <0.001 |
| Computer Science | men | 85.41 ± 7.66 | 81.21 ± 11.99 | 4.20 (-0.01- 8.40） | 1.984 | 0.050 |
|  | women | 82.77 ± 8.19 | 83.72 ± 9.10 | -0.95 (-2.95- 1.06） | -0.928 | 0.354 |

Table 4 Academic achievements comparison of foundational course between the pre-COVID-19 cohort and COVID-19 cohort ($\bar{x}\pm s$)

| Courses | pre-COVID-19 cohort (n=195) | COVID-19 cohort (n=180) | mean difference, 95% CI | *t* value | *P* value |
| --- | --- | --- | --- | --- | --- |
| Physiology | 71.51 ± 7.42 | 71.75 ± 7.20 | -0.24 (-1.73- 1.25) | -0.318 | 0.751 |
| Biochemistry | 77.52 ± 7.67 | 68.53 ± 6.67 | 8.99 (7.53- 10.45) | 12.128 | <0.001 |
| Pathoimmunology | 74.94 ± 6.85 | 78.97 ± 8.06 | -4.02 (-5.55- -2.50) | -5.188 | <0.001 |
| Physiological Practice | 82.13 ± 4.96 | 88.92 ± 2.97 | -6.79(-7.61- -5.97) | -16.218 | <0.001 |

Table 5 Academic achievements comparison of foundational course between the pre-COVID-19 cohort and COVID-19 cohort based on sex stratification ($\bar{x}\pm s$)

| Courses | Group | pre-COVID-19 cohort (n=195) | COVID-19 cohort (n=180) | mean difference, 95% CI | *t* value | *P* value |
| --- | --- | --- | --- | --- | --- | --- |
| Physiology | men | 69.20 ± 7.46 | 69.87 ± 7.41 | -0.66 (-3.86- 2.53） | -0.413 | 0.680 |
|  | women | 72.28 ± 7.28 | 72.25 ± 7.09 | 0.29 (-1.64- 1.70) | 0.035 | 0.972 |
| Biochemistry | men | 75.22 ± 7.44 | 65.92 ± 6.00 | 9.30 (6.44- 12.17) | 6.454 | <0.001 |
|  | women | 78.30 ± 7.62 | 69.23 ± 6.69 | 9.06 (7.40- 10.72) | 10.733 | <0.001 |
| Pathoimmunology | men | 71.96 ± 6.35 | 75.80 ± 7.55 | -3.84 (-6.81- -0.88) | -2.579 | 0.012 |
|  | women | 75.95 ± 6.74 | 79.82 ± 8.01 | -3.87 (-5.59- -2.16) | -4.441 | <0.001 |
| Physiological Practice | men | 78.33 ± 5.04 | 88.58 ± 3.35 | -10.25 (-12.14- -8.37) | -10.811 | <0.001 |
|  | women | 83.40 ± 4.24 | 89.00 ± 2.86 | -5.60 (-6.44- -4.77) | -13.169 | <0.001 |

Table 6 Academic achievements comparison of Nursing essential course between the pre-COVID-19 cohort and COVID-19 cohort ($\bar{x}\pm s$)

| Courses | pre-COVID-19 cohort (n=195) | COVID-19 cohort (n=180) | mean difference,  95% CI | *t* value | *P* value |
| --- | --- | --- | --- | --- | --- |
| TCM Nursing | 82.08 ± 6.34 | 86.65 ± 7.05 | -4.56 (-5.93- -3.20) | -6.582 | <0.001 |
| Combined Nursing | 74.11 ± 7.16 | 82.77 ± 6.53 | -8.65 (-10.05- -7.26) | -12.201 | <0.001 |

Table 7 Academic achievements comparison of Nursing essential course between the pre-COVID-19 cohort and COVID-19 cohort based on sex stratification ($\bar{x}\pm s$)

| Courses | Group | pre-COVID-19 cohort (n=195) | COVID-19 cohort (n=180) | mean difference,  95% CI | *t* value | *P* value |
| --- | --- | --- | --- | --- | --- | --- |
| TCM Nursing | Male | 79.35 ± 5.90 | 86.08 ± 9.28 | -6.73 (-10.00- -3.47) | -4.103 | <0.001 |
|  | Female | 83.01 ± 6.23 | 86.79 ± 6.38 | -3.78 (-5.25- -2.32) | -5.087 | <0.001 |
| Combined Nursing | Male | 69.80 ± 5.23 | 83.00 ± 5.95 | -13.20 (-15.59- -10.82) | -10.994 | <0.001 |
|  | Female | 75.56 ± 7.15 | 82.70 ± 6.69 | -7.14 (-8.75- -5.54) | -8.750 | <0.001 |

Discussion
*---The beginning of the discussion should be with the purpose of study*

We have inserted an additional paragraph at the beginning of the Discussion summarising the results in terms of the stated aims, it now reads: “The current study has shown that the impact of the pandemic-driven change in teaching delivery to a purely online mode of teaching has not detrimentally impacted nursing student early learning, and, indeed, has substantially improved student learning in several subjects. Taken together, these data suggest that more extensive online learning methodologies should be incorporated into nursing training, although face-to-face learning still has a beneficial role in certain complex subjects, such as Biochemistry.” (Discussion, page 8, first para, 253-258)

*The researcher can look for more arguments and bring his own argument in the discussion. For example, maybe the reason for the difference in the number of male and female students is the effort and study of women to enter this field.*

We apologise for this confusion. The discussion about difference between men and women students have been presented: “Surprisingly, there was no obvious difference in the scores in Physiology between male and female students in both 2019 and 2020, nor the general performance between 2019 and 2020. We speculate that the new students would have a general understanding of biology from their high school studies which may offer a bridge for the transition from high school biology to Physiology. Our speculation is supported by the finding that no significant difference was observed between male and female medical students in Physiology [19]. This explains the similar performance of both males and females in their Bachelor course and also the consistency of the overall scores between 2019 (traditional lectures) and 2020 (online teaching). (Discussion, page 8, last para, 300-307).”

*The researcher can look for more arguments and bring his own argument in the discussion. For example, maybe the reason for the difference in the number of male and female students is the effort and study of women to enter this field*.

*---In discussion explain the important results of similar studies and interpret the differences.*
To address this point we have added the following sentences in the Discussion, it now reads: “Notably, in several subjects, women students performed better than men students both pre-COVID‑19 and/or during the lockdown. The students’ performance might be influenced by a number of factors e.g. conscientiousness and self-motivation [22], which is consistent with the findings from others, showing that performance by women is better in tertiary health subjects [18]. The difference was thought to be a result of increased self-efficacy from women students. Thus, the perception from women students was that they possess a higher level of confidence, which motivated them to complete the required learning tasks for their course, and subsequently led to greater mental effort and persistence, compared to men students.” (Discussion, page 9, last para 347-354)

Conclusion
*--- Explain more about the message you want to deliver in students support mental health.*

Furthermore, it has been demonstrated that there has been significantly increased levels of depression and anxiety among the Chinese students and other medical staff; while economy staffs are highly stressed at the end of the lockdown in China [25]. These findings are supporting our current hypothesis that there is potential high risk contributing to impairment of psychological status of these students with serious and even potentially irreversible outcomes. These impacts of the COVID-19 pandemic will be explored in future studies. (Discussion, Page 10, first para, lines 370-375).

***Reviewer #2***

*I would suggest the authors to edit their work for grammar, consistency and in some pages, particularity at the conclusion to rewrite so that readers can get the meaning out of it. There is need to include the role of the overall study in a form of implication or recommendation.*

We have modified our manuscript accordingly.

**Reviewer #3**

The manuscript is written professionally, reading it was a pleasure. I have very minor comments to address:

*- In the Abstract and Methodology sections the numbers of students (participants) in each cohort are not mentioned. The authors need to shed some light on this*.

We have modified accordingly, it now reads: “There were 195 students in pre-COVID-19 cohort, including 49 men and 146 women. And there were 180 students in COVID-19 cohort, consisting of 38 men and 142 women.”

*- Some of the paragraphs (under the Introduction section) are very short and can be merged. For example, the second and third paragraphs can be merged. This is simply because academic writing does not favour very short paragraphs.*

We have modified accordingly.

*- On the same page (pages are not numbered, but I assume page 9), line 100, it is mentioned that “Our University School of Medicine not did not decline”. Here “not” is repeated*

We have removed the extra "not".

*- Also, on the same page, line 102, it is mentioned that “(manuscript is currently being reviewed).” Here, check journal appropriate referencing style for referring to an under-review manuscripts.*
We have replaced the published manuscripts with our manuscript under review, it now reads: “It has been reported that the mental health is significantly harmed, e.g. increased stress level, including fear of infection and isolation following long-term lockdown [12] among general populations, and anxiety among medical staffs and medical students, perhaps due to excessive media consumption was linked to mood disorders during the lockdown in China [13, 14]. However it remains to be explored the impact of lockdown in the training of nursing students. It is well known the importance of training competent nursing students, who would become specialized registered nurses and play critical role in the medical practices either in hospitals and/or private practices. On the other hand, incompetent nurses would compromise at the different levels of outcomes for the management of patients.” (Introduction, page 3, last para lines 104-112).

*- Conclusion section: Check spelling of the word ‘excise’ in the phrase “Physical excise” (line 349).*
We have modified the word, from ‘excise’ to ‘exercise’.

We hope our revision is sufficient for publication in Plos One.

Please let us know if you have further queries.

Yours sincerely

Shisan Bao
